# Adult ADHD: Questioning Diagnosis and Treatment in a Patient with Multiple Psychiatric Comorbidities

**DOI:** 10.1155/2017/1364894

**Published:** 2017-06-11

**Authors:** Robert Karoly Chu, Tea Rosic, Zainab Samaan

**Affiliations:** ^1^Michael G. DeGroote School of Medicine, McMaster University, Hamilton, ON, Canada; ^2^Department of Psychiatry and Behavioural Neurosciences, McMaster University, Hamilton, ON, Canada

## Abstract

Adult Attention-Deficit/Hyperactivity Disorder (ADHD) is a contentious diagnostic issue, which has been increasing in prevalence in recent years, and is often comorbid with other psychiatric disorders. This report presents a detailed account of a clinical case involving a middle-aged man with a history of recurrent depressive episodes and an unsubstantiated diagnosis of ADHD, treated with stimulants. There is persistent debate around the use of psychostimulants both in adult ADHD and in the treatment of depression. Despite promising activating properties, psychostimulants carry significant risks of misuse and substance use disorder. In this report, we consider the potential benefits and adverse effects of stimulants in the treatment of adult ADHD and mood disorders and review the learning points of this complicated, but not uncommon, clinical case.

## 1. Introduction

There has been a considerable increase in diagnosing adults with Attention-Deficit/Hyperactivity Disorder (ADHD) and subsequent use of psychostimulants [1]. These medications have also been used for other adult psychopathologies, including depression, based on weak evidence for short-term use and lack of data on developing stimulant use disorder [2]. Prescribing data suggest that the use of psychostimulants in adults has increased significantly in the last two decades in both the United States [3] and the United Kingdom [4].

ADHD is characterised by persistent inattention and/or hyperactivity and impulsivity beginning before age of 12 and causing impairment in two or more settings [5]. Although ADHD assessment in adults and the evidence for adult onset of ADHD remain debated, treatment with psychostimulants is common, and patients are invested in the diagnosis and continued treatment. Major depressive disorder (MDD) is characterised by depressed mood and fatigue, among other symptoms [5], and it is not unusual for effective treatment to necessitate trials of many different pharmaceutical agents [6]. Psychostimulants, such as methylphenidate and amphetamine, have activating properties. Hence, it is intuitively possible that these agents may have a role in the treatment of MDD. In both cases, an examination of the evidence surrounding psychostimulants in clinical practice is important to address. Specifically, we aim to

1. present a detailed account of a clinical case of a middle-aged man with MDD and unsubstantiated adult ADHD,
2. review the diagnosis of ADHD in adulthood,
3. consider the adverse effects of psychostimulants and their potential for causing substance use disorders,
4. examine the evidence regarding psychostimulants in the management of MDD in both general and specific patient populations.

Written informed consent to present this case was obtained from the patient prior to writing this report.

## 2. Case Presentation

A 62-year-old man was brought to the Psychiatric Emergency Service (PES) after reporting suicidal ideation to a local crisis outreach team. Initial assessment revealed a three-week history of increasingly depressed mood, culminating in contemplation of suicide with a plan to cut his wrists on the day of presentation. He endorsed intrusive negative thoughts, irritability, and increased sensitivity to sound. He denied both psychotic symptoms and recent substance use.

Collateral history from his wife revealed a history of previous psychiatric admissions, but there were none in the past decade. She reported that, over the last two months, the patient had been socially isolated, irritable, despondent, and hopeless. She attributed this exacerbation to the stressors of deteriorating vision impairing the patient's ability to engage in hobbies, recent medication changes, and an altercation with a neighbour.

Mental status examination in PES revealed a middle-aged man dressed in nightwear and appearing younger than his stated age. He was lying in bed, turned away, and avoiding eye contact. His motor movements were slowed. He was cooperative in answering questions. Speech was soft, monotone, and somewhat slurred. His thought process was organised and coherent. Thought content was notable for negative ruminations. There were no apparent perceptual disturbances. Mood was low and affect flat. Cognition, while not formally assessed, was grossly intact. He showed reasonable insight and judgment.

The patient was admitted to inpatient psychiatry on a voluntary basis for stabilisation and medical reassessment. On admission, he was quite agitated, voicing concerns of alleged maltreatment by staff and demanding to go to another hospital. When settled, he reported having been depressed since childhood and denied any history of suicide attempts or intent to harm himself at the time of admission, which differed from the initial PES assessment.

The patient lives independently with his wife. They have three adult children. He completed his high school education and went on to college. He has been collecting disability benefits since 1993, when his deteriorating vision made it no longer possible for him to work. He endorsed use of tobacco, while denying current use of alcohol. He does have a history of alcohol use disorder but has been abstinent for the last 20 years. He endorsed regularly smoking cannabis but denied recent use. Family history is positive for depression. He has a legal history pertaining to marijuana possession and an assault charge. Documentation also notes a history of kleptomania but does not elaborate.

His psychiatric history dates back to 1993, with three past psychiatric admissions, most recently in 2001. For the past 12 years, he had been seen as an outpatient in a psychiatry clinic where medication trials included citalopram, fluoxetine, venlafaxine, reboxetine, phenelzine, tranylcypromine, mirtazapine, lamotrigine, perphenazine, and diazepam. Also, he had tried psychotherapy for depression and a trial of ten electroconvulsive therapy (ECT) treatments in 1995 with no benefit. When his perphenazine was tapered in 2004, due to concerns of tardive dyskinesia, his mood deteriorated and guilty ruminations worsened. His mood had become significantly destabilised in 2014 following a five-day course of prednisone for epiglottitis that had resulted in an isolated self-reported “manic” episode, although documentation does not elaborate on what this entailed. Records note that the patient has historically had difficulty accepting psychological factors as contributors to his depression and that he has focused on pharmacotherapy.

The patient was diagnosed with ADHD as an adult, prior to 2001, though the exact date could not be determined. Records indicate that he had difficulties with concentration even when his other depressive symptoms were alleviated, but there was no documentation of other symptoms of ADHD. The patient himself was not able to provide history consistent with ADHD symptoms in childhood. In 2001, he was taking methylphenidate (extended-release 40 mg twice daily and immediate-release 10 mg twice daily) as treatment for ADHD, with improvement in concentration, and for augmentation of treatment for his mood disorder. However, he later developed “psychological and physical dependence” on methylphenidate, and therefore this was discontinued and temporarily replaced with clonidine. In 2005, he began atomoxetine (25 mg), a nonstimulant for ADHD, which he was tolerating well at 40 mg daily.

Chart review demonstrated previous diagnoses of social anxiety disorder and generalised anxiety disorder, as well as features of narcissistic, antisocial, and borderline personality disorders. Past medical history also included glaucoma and legal blindness, migraines, dyslipidaemia, obstructive sleep apnoea, gastrooesophageal reflux disease, and renal cell carcinoma treated by partial nephrectomy in 2004. Preadmission medications included amphetamine 30 mg twice daily, lurasidone 40 mg daily, mirtazapine 60 mg every bedtime, perphenazine 4 mg twice daily, and quetiapine 300 mg every morning and 700 mg every bedtime. On an as-needed basis, he was prescribed diazepam 10 mg twice daily. The patient was also prescribed brimonidine-timolol 0.2/0.5% 1 drop twice daily and budesonide 64 mcg 2 sprays each nostril daily. Over the two months prior to his current presentation, he was in the process of tapering paroxetine (60 to 20 mg) and adding vortioxetine (10 to 20 mg), while also attending a 13-week cognitive-behavioural therapy group for depression.

Over the course of his admission, the patient's suicidal ideas subsided, but he continued to report intrusive negative thoughts, low mood, and feelings of worthlessness. His behaviour was highly variable, sometimes from hour to hour. At times, he was disrespectful and aggressive; at others, he was pleasant and apologetic for past behaviour.

## 3. Investigations

Laboratory investigations on admission, including complete blood count, electrolytes, creatinine, fasting glucose, and lipid panel, were within normal range. Urine drug screen was positive for amphetamines, benzodiazepines, and THC.

A baseline ECG was unremarkable. A brain MRI ordered to rule out organic pathology demonstrated two 12 mm meningiomas with unclear clinical significance. Upon consultation with the neurology service, a repeat MRI was requested two months later due to concern about metastases related to the patient's past history of renal cell carcinoma. The follow-up MRI completed two months later demonstrated interval stability of the previously seen meningiomas and no evidence of new pathology, with recommended repeat MRI in one year.

## 4. Differential Diagnosis

The patient had an extensive history of MDD managed with myriad pharmacologic agents, as well as ECT. His current presentation was consistent with those documented in his past. Furthermore, he had no history of manic symptoms, aside from one episode apparently induced by corticosteroids, making the possibility of bipolar disorder remote. Clinical diagnostic clarification was done to rule out social anxiety disorder, generalised anxiety disorder, and personality disorders, based on the multiple past diagnostic labels in the chart, and showed no clear evidence for any of these diagnoses.

The issue, then, was to clarify the validity of his ADHD diagnosis and consequent treatment with amphetamine. Aside from anecdotal concentration difficulties, the patient's presentation was devoid of other ADHD symptoms, and his documented medical history showed the same, allowing us to reliably rule out ADHD. Other possible causes for poor concentration include his primary MDD versus a drug-induced phenomenon secondary to polypharmacy.

## 5. Treatment

In this patient, significant polypharmacy must be considered for potential drug interactions. For example, mirtazapine may increase serotonergic effects when given with a serotonin stimulator such as vortioxetine and therefore may increase patient's symptoms of irritability and impulsivity. While the combination of antipsychotics is likely to potentiate side effects and in this particular case the suicidal behaviour, it has been reported that quetiapine and lurasidone may be associated with suicidal behaviour and therefore the patient's initial presentation may also be explained by polypharmacy drug interactions. To reduce polypharmacy, certain psychotropic medications were tapered: mirtazapine was reduced to 45 mg every bedtime, lurasidone to 20 mg every bedtime, perphenazine to 2 mg twice daily, and quetiapine to 100 mg every morning and 600 mg every bedtime. Vortioxetine was discontinued. The patient was on three antipsychotic medications with no history of psychotic disorder; therefore, lurasidone and perphenazine were tapered and discontinued.

Given the lack of support for a diagnosis of ADHD, risk of cardiac adverse effects, and the patient's aggression, impulsivity, and mood variability over his admission, we suggested that he discontinue amphetamine. Initially, he was resistant, attributing amphetamine to improving his concentration and mood, stating that it “is the only thing that makes me feel good.” After two weeks of admission, he was amenable to making the change, stating that he felt amphetamine made him “shaky,” that it might not be helpful, and that he would like to reduce the total number of medications he was taking. Therefore, amphetamine was tapered by a reduction of 10 mg daily every three days, which he tolerated well. An excerpt of the patient's perspective can be found below.

The patient was also trialed on clomipramine, as he had never been trialed on a tricyclic antidepressant. In consultation with ophthalmology, a trial of clomipramine was deemed acceptable despite previous glaucoma, as the condition had resolved with surgery. The patient initially tolerated clomipramine but developed tinnitus after three weeks, and so the medication was discontinued. He was started on venlafaxine, to which he had a good response for several years in the past.

To optimize his medical management, we added ASA 81 mg daily, brinzolamide 1% topical twice daily, ranitidine 300 mg daily, and vitamin D 1000 IU daily. On an as-needed basis, the following medications were added: acetaminophen 325–650 mg every four hours, ibuprofen 200–400 mg every four hours, naproxen 250–500 mg every six hours, docusate sodium 100 mg twice daily, lactulose 30 mL daily, senna 17.2 mg at bedtime, nicotine 10 mg inhaler cartridge every one hour (to help with smoking cessation), benztropine 1-2 mg every one hour, lorazepam 0.5 mg every four hours, and zopiclone 7.5 mg at bedtime.

## 6. Outcome and Follow-Up

The patient was discharged after 65 days in hospital. At that time, his mood had improved and he was tolerating the medication adjustments. Discharge medications included the following: quetiapine 100 mg in the morning and 600 mg every bedtime, venlafaxine 187.5 mg daily, to be increased to 225 mg daily two days after discharge, melatonin 3 mg every bedtime, ranitidine 300 mg daily, vitamin D 1000 IU daily, brimonidine-timolol drops, brinzolamide drops, budesonide spray, docusate sodium, and lactulose. On an as-needed basis, he was provided quetiapine 50 mg up to three times daily for one month, zopiclone 7.5 mg every bedtime for one month, and 60 tablets of lorazepam 1 mg up to twice daily.

Follow-up appointments were arranged with the patient's family physician, outpatient psychiatrist, and outpatient neurologist. The patient agreed to begin individual mindfulness sessions with a psychology resident and to begin attending a weekly emotion regulation skills group.

## 7. Discussion

### 7.1. Diagnosis and Treatment of ADHD

ADHD is a neurodevelopmental disorder in DSM-5 [5], usually first diagnosed in childhood. Changes to the diagnostic criteria have been made to assist in the diagnosis of adults with the disorder, such that only five, rather than six, of either the inattentive or hyperactive-impulsive criteria are required for diagnosis.

Although previously thought to remit in adulthood [7], 65% of those diagnosed with ADHD as children show persistent symptoms as adults, and the prevalence of the disorder in adults is estimated at 2–5% [8]. Though adults may have difficulty recalling childhood symptoms [5], adult onset is not thought to occur [9]. With age, motor hyperactivity becomes less prominent, while more subtle symptoms like inner restlessness, impulsivity, and inattention remain [5, 8]. These symptoms affect work performance and attendance, impair relationships, and increase the likelihood of injury, traffic accidents, and obesity [5]. Hence, effective treatment is critical to reducing impairment in multiple functional domains.

ADHD symptoms likely respond just as well to stimulants in adults as in children [10], with 70% of patients reported to benefit from psychostimulants [8, 11]. Should an adequate response be achieved, therapy may continue indefinitely, so long as it remains effective, and is reviewed on an annual basis [12].

While our patient felt that amphetamine improved his concentration and ability to complete tasks, his diagnosis of ADHD is questionable as chart review and clinical history failed to substantiate the diagnosis. Importantly, a positive response to stimulants does not validate the diagnosis of ADHD [1]. Given that psychostimulants reduce the severity of symptoms but do not necessarily eliminate them, we expected to see some evidence of the disorder during this admission, but none was observed. Taken together, we presume that his diagnosis of ADHD was inaccurate at the time of presentation.

### 7.2. Adverse Effects and Abuse Potential of Psychostimulants

While psychostimulants are generally well tolerated [13], they are associated with numerous side effects, including headache, reduced appetite, weight loss, palpitations, anxiety, insomnia, tics, xerostomia [8, 12], and agitation [14, 15]. Increases in heart rate and blood pressure, although clinically insignificant [16], warrant monitoring [12]. No research has been done on the long-term effects of stimulant treatment over years or decades in adult patients [1]. Moreover, hypertension, cardiac angina, hypertrophic cardiomyopathy, cardiac arrhythmia, hyperthyroidism, and, importantly for our patient, glaucoma are considered relative contraindications to psychostimulant therapy [8]. A randomised controlled trial (RCT) of mixed amphetamine salts revealed similar side effect profiles in adults to those reported in children [17]. In addition, no clinically significant changes in vital signs or ECG were found [17]. When used in the context of bipolar disorder, for treatment of ADHD or depression, there has been concern that psychostimulants may induce switching to a manic state [18]; however, this has not been found in studies to date [14, 19, 20].

Psychostimulants are chemically and pharmacologically similar to drugs of abuse, like cocaine and amphetamine [21, 22], and have euphorigenic properties [20]. In fact, laboratory studies in both humans and animals have clearly shown potential for abuse of methylphenidate, although there is little evidence to support this as a concern in individuals with ADHD prescribed the drug [23, 24]. While psychostimulants may find illicit use in the pursuit of euphoria, they are also used as performance enhancement agents [25] as a result of their ability to improve concentration and focus. However, the nonmedical use of psychostimulants is low, about 2% in American adults, the vast majority of whom do not have a legitimate prescription [26]. Although there has been an increase in the number of prescription stimulants issued in USA for patients without a diagnosis of ADHD [3], a 2007 review suggested that, at the doses used in the treatment of depression, psychostimulants are not addictive [20]. The long-acting, slow-release formulations of psychostimulants maintain more stable drug levels than the short-acting forms, putting them at further reduced risk for abuse or dependence [13, 27].

Our patient had a history of glaucoma, substance-induced mania, and anxiety, any of which could have been exacerbated by psychostimulant treatment. In addition, over the course of his admission, he demonstrated mood lability and agitation, which may also be attributed in part to amphetamine therapy. Given the patient's decade-long history of amphetamine use, previous addiction to methylphenidate, and his past history of alcohol use disorder, amphetamine tolerance and dependence were also concerns. The patient insisted that he only ever took the drug as prescribed and did not exceed the recommended dose. However, in this case, there is no evidence supporting a diagnosis of ADHD, the primary indication for amphetamine. Further, the patient had previously developed a substance use disorder to methylphenidate, and his current presentation of changing behavior is likely stimulant-related. This case highlights how stimulants may not be as benign as reported in the literature. The patient did have a significant history of depression, and therefore we considered how depression might be an indication for his psychostimulant prescription.

### 7.3. Psychostimulants in the Treatment of Depression

Stimulants were first used in the treatment of depression in the 1930s but were eclipsed by tricyclic antidepressants in the 1950s, which stated to have no potential for abuse, tolerance, or dependence [14]. Currently, evidence-based guidelines for the treatment of depression assert that antidepressants are first-line therapy for moderate-to-severe depression [28]. However, with traditional antidepressant therapy of appropriate trial duration and dose optimisation, only 50–60% of patients will have an adequate response, with just 35–40% achieving remission [29]. In excess of 60% of patients suffering from a depressive episode will relapse [14], making depression a chronic, disabling disorder. One-fifth of patients will show resistance to conventional pharmacotherapy [14], making a place for alternatives.

In cases of treatment failure or resistance, the recommended strategy is to increase the dose, change antidepressants, or augment therapy [28]. Electroconvulsive therapy (ECT) should be considered in cases of treatment-resistant depression [30]: our patient had received ten ECT treatments in the past, albeit without improvement. Augmentation with another psychotropic medication may be considered if there has been a partial response with good tolerability to the current antidepressant [28]. Recommendations for augmentation suggest aripiprazole, quetiapine, or lithium as first-line choices, followed by olanzapine, risperidone, mirtazapine, and triiodothyronine as second-line options [28].

Because psychostimulants have euphorigenic and alerting properties [20], there has been interest in applying them in augmentation of treatment for depression. A 2013 study examining patients who had trialed, on average, five antidepressants and then added either dexamphetamine or methylphenidate to augment current therapy demonstrated at least some level of benefit in 50% of cases [31]. However, this may simply be a result of stimulants reducing fatigue and apathy, rather than depressive symptoms [32, 33]. An RCT that added extended-release methylphenidate as an augmentation strategy in treatment-resistant depression showed no advantage over placebo [34]. Another study that compared sertraline therapy augmented with methylphenidate to sertraline alone failed to demonstrate a significant difference in outcomes [35]. Hence, while stimulants have been used clinically in treatment-refractory depression, there is little evidence to support them in therapy augmentation. Current guidelines note that the evidence in support of stimulants in antidepressant augmentation is only of grade “C” strength [28]. In the general population, psychostimulants may not have a role in depression, given the lack of evidence for either monotherapy or augmentation and the small but present concerns of adverse effects and addictive potential.

Our patient had previously been trialed on atomoxetine, a nonstimulant medication used in the treatment of ADHD [36]. Atomoxetine has been studied for its potential in augmenting treatment for depression, as it is thought to have no abuse potential and to cause less insomnia, although its onset is slower and its effectiveness is inferior in treating ADHD [36]. Case reports and small studies have demonstrated clinical improvement with atomoxetine added to selective serotonin reuptake inhibitor (SSRI) therapy for partially responsive major depression [33–39]. However, an RCT of atomoxetine augmentation for patients partially responsive to sertraline therapy did not show a beneficial response over placebo [40].

There are certain populations for which stimulants may be a preferred treatment for depression, particularly palliative and elderly patients [41–44]. Fatigue is a frequent physical symptom of depression and medical illness [44]. There is ample evidence that psychostimulants alleviate fatigue [15, 20, 44], and this has been shown to occur in a dose-dependent fashion [45]. Perhaps the greatest advantage of stimulants is that they work quickly: their onset of effect is generally within 48 hours [46, 47], while conventional antidepressants take several weeks to reach efficacy [46]. It is thought that psychostimulants potentiate the effect and cover the therapeutic latency period of conventional antidepressants [48]. Small studies of citalopram augmentation with methylphenidate in geriatric depression revealed more rapid improvement with combination therapy over either drug alone [49, 50]. Patients near the end of life who suffer from depression stand to gain more from the rapid alleviation of their symptoms than the general population. Although considered safe in these populations, as many as 10% may have adverse effects warranting discontinuation of therapy [46], and the potential elevations in heart rate and hypertension must be considered carefully [51]. Hence, while there is insufficient evidence to recommend psychostimulants in treating the general population for depression, they may warrant consideration for specific subsets of the population.

## 8. Conclusion

Considering the paucity of evidence to support use of stimulants in routine treatment of depression and the fact that this patient did not meet DSM-5 criteria for ADHD, there was little to justify his continued use of a stimulant. Hence, with his approval and consent, we tapered and then discontinued his amphetamine.

## Additional Points

*Learning Points*. (i) ADHD in adults is common, affecting 2–5% of the adult population [3]. DSM-5 diagnostic criteria do require some symptoms to have been present prior to age of 12 [1].

(ii) Psychostimulants are first-line treatment for ADHD [3], but there is insufficient evidence to recommend their use either as first-line or as adjunctive therapy for MDD in the general population.

(iii) Psychostimulants are generally well tolerated, and concerns regarding cardiovascular adverse effects are negligible [8]. While addictive potential is an important consideration, there is little evidence that dependence and abuse are a major problem [17, 18].

(iv) In treating MDD in the general population, there is insufficient evidence to recommend psychostimulants as either primary or adjunctive pharmacotherapy [14, 21, 31, 32]. In the elderly and those who are terminally ill, their rapid action in alleviating fatigue and depressive symptoms make them especially useful [38].

*Patient Perspective*. “If I were on heroin, I'd be better understood. I'm disliked for being on Adderall. They're fixated on me not being on Adderall. I was first diagnosed [with ADHD] 25 years ago. When I was young, I know now, I suffered from depression. All through school, I could not concentrate. They thought this guy is stupid. Went through childhood being labeled slow. My concentration was very low. When you're there at 16-years-old, you don't get along with people. One tends to take up drugs and alcohol, and that's what I did. I was diagnosed at 38. Immediately, I was treated for depression. When they started treatment for ADD, I was given Ritalin. It's a real nasty drug. There's a crash—a sense of depression. You go up and you go down—within hours. I was on it for three years. It was the best they could do for me. Ten years ago, my doctor tried a course of Adderall. I took the Adderall, my ADD went away instantly. No more psych patient. Out and about, doing things. They're taking me off—will be sitting on my butt. When you're sick for a long time, you'll do anything. I did ECT. I'm hurting again now. I'll take a short good life over a long suffering life. I'm about to cry—I'm so depressed. Adderall helps with depression too—it's minimized by it. The way I see it, they want to take my life away. But, you see, for me, it's life and death—I could kill myself if I see myself sitting around in front of the TV for six months. It really helped the mood. I could sleep at night—it didn't disturb my sleep. I didn't feel I was on a drug at all. It probably suppresses the appetite a bit. If you could take me off Adderall and I'd be functioning well, then great—that's one less drug. I feel like it's the only thing that ever worked. If I could be happy without it, that'd be great. I get the feeling I'm being treated like an addict. I'm an alcoholic—everyone in my family is. Was it addictive? Yeah. But I stopped it. I can stop things. I know it's addictive, because I just stopped it—and the doctors said you have to go off slowly. I never took more than prescribed. Ten years on Adderall—it was great. Moved, bought a house, fixed it up. I was fully functioning.”
